# Novel Splicing Variant in the *PMM2* Gene in a Patient With PMM2-CDG Syndrome Presenting With Pericardial Effusion: A Case Report

**DOI:** 10.3389/fgene.2020.561054

**Published:** 2020-10-07

**Authors:** Katerina Slaba, Hana Noskova, Petra Vesela, Jana Tuckova, Hana Jicinska, Tomas Honzik, Hana Hansikova, Petra Kleiblova, Petr Stourac, Petr Jabandziev, Ondrej Slaby, Dagmar Prochazkova

**Affiliations:** ^1^Department of Pediatrics, University Hospital Brno, Faculty of Medicine, Masaryk University, Brno, Czechia; ^2^Central European Institute of Technology, Masaryk University, Brno, Czechia; ^3^Department of Pediatrics and Inherited Metabolic Disorders, First Faculty of Medicine, Charles University and General University Hospital in Prague, Prague, Czechia; ^4^Institute of Biology and Medical Genetics, First Faculty of Medicine, Charles University and General University Hospital in Prague, Prague, Czechia; ^5^Department of Pediatric Anaesthesiology and Intensive Care Medicine, University Hospital Brno, Faculty of Medicine, Masaryk University, Brno, Czechia; ^6^Department of Biology, Faculty of Medicine, Masaryk University, Brno, Czechia; ^7^Institute of Medical Genetics, Faculty of Medicine, Masaryk University, Brno, Czechia

**Keywords:** PMM2-CDG, pericardial effusion, whole exome sequencing, novel splicing variant, phosphomannomutase 2

## Abstract

Congenital disorders of glycosylation (CDG) are a rapidly growing family of genetic diseases with the phosphomannomutase 2 (PMM2)-CDG being the most common form of CDG. Most of these monogenic diseases are autosomal recessive and have multi-systemic manifestations, mainly psychomotor retardation, facial dysmorphisms, characteristic distribution of the fat pads, and variable coagulation abnormalities. The association of fetal hydrops with CDG has been reported, and pericardial effusion was also rarely observed in patients with PMM2-CDG. Here we describe an infant boy with PMM2-CDG. The diagnosis was suspected based on inverted nipples, fat pads, and combined coagulopathy. However, the primary symptom was progressive pericardial effusion leading to patient death at the age of 3 months. Screening for CDG performed by the use of isoelectric focusing of serum transferrin showed a typical PMM2-CDG pattern. Exome sequencing revealed one common pathogenic variant (c.691G > A/p.Val231Met) and one novel variant (c.447 + 3dupA) in the *PMM2* gene. Both *PMM2* variants were further confirmed by Sanger sequencing in both the proband and the parents’ DNA. The novel variant was predicted to result in loss of donor splice site, and the analysis at mRNA level confirmed that it leads to exon five skipping (r.348_447del) and causes premature termination of translation to the protein (p.G117Kfs^∗^4), therefore is classified as likely pathogenic. Although there is no curative therapy for the PMM2-CDG at the moment, the other supportive care options are available to be offered. The definite diagnosis of PMM2-CDG can also assist in the process of genetic counseling, family planning, and preimplantation genetic diagnosis.

## Introduction

Congenital disorders of glycosylation (CDG) are a genetically and clinically heterogeneous group of > 130 disorders characterized by genetic defects in the synthesis and attachment of glycoprotein and glycolipid glycans (Chang et al., 2018). The most common form of CDG is phosphomannomutase 2 (PMM2)-CDG (formerly known as CDG-Ia) (OMIM 212065), a multisystem disease with a wide range of clinical presentation and the phenotype ranging from mild adulthood form to very severe neonatal form (Peanne et al., 2018). PMM2-CDG is an autosomal recessive disorder, caused by mutations in the PMM2 gene localized on chromosome 16p13 (MIM 601785), with the prevalence ranging from 1/20,000 in Dutch populations and 1/77,000 in Estonia based on isolated reports (Schollen et al., 2000; Vals et al., 2018).

From the pathophysiological point of view, PMM2-CDG is a disorder of protein N-glycosylation characterized by genetic defects leading to deficiency/dysfunction of PMM2, the enzyme responsible for the conversion of mannose-6-phosphate into mannose-1-phosphate (Matthijs et al., 2000). Mannose-1-phosphate is a precursor of guanosine diphosphate mannose (GDP-Man) and dolichol-P-mannose (Dol-Man). Deficiency of GDP-Man and Dol-P-Man causes hypoglycosylation of numerous glycoproteins, including serum glycoproteins (lysosomal enzymes and transport proteins) and membrane glycoproteins. This results in multi-organ involvement, whereas the variability of disease severity and course are not fully understood (Altassan et al., 2019).

Here, we describe a novel splicing mutation in a case of PMM2-CDG, presented with pericardial effusion, with typical dysmorphic facial features, inverted nipples, failure to thrive, and psychomotor retardation.

## Case Presentation

A Czech boy was born as a first child of unrelated parents. He was diagnosed with hemodynamically insignificant pericardial effusion in the 22^nd^ week of pregnancy. Amniocentesis was performed with no evidence of aneuploidy. There was an aberration of unknown significance Xp22.33 × 3 and three copies of the CYP21A2 gene in combination with a pathogenic variant p.Gln319^∗^ detected hypothetically associated with congenital adrenal hyperplasia; however, the determination for 17-hydroxyprogesterone in amniotic fluid provided a negative result. Routine testing of the mother for toxoplasmosis, parvovirus B19, CMV, and HSV infections was negative. Family history was unremarkable. Labor was induced in 40 weeks of pregnancy for intrauterine growth retardation and oligohydramnios. Delivery was by forceps-assisted vaginal delivery due to fetal heart rate deceleration. A birth weight of the boy was 2390 g (*z*-score = −1.83), length of 48 cm, and occipital frontal circumference 34 cm. His postnatal adaptation was normal, but he was noted to be highly dysmorphic. Initially, due to swallowing difficulties nasogastric tube was used for feeding.

**FIGURE 1:**
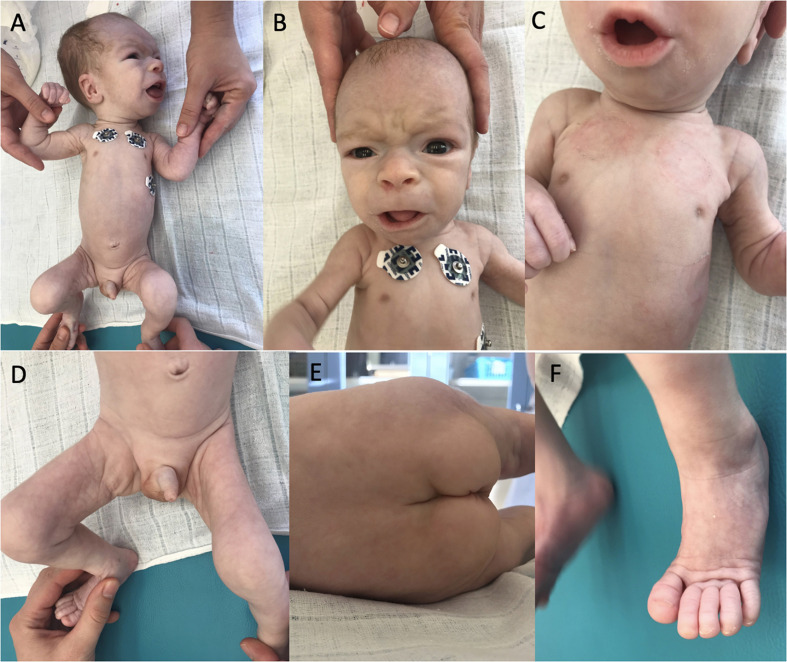
Dysmorphic features of our patient. Dolichocephaly, bossing forehead, dysmorphic low set ears **(A)**, facial dysmorphism **(B)**, bilateral inverted nipples **(C)**, abnormal fat distribution **(D,E)**, and hammertoes **(F)**.

At the age of 4 weeks pericardial effusion further progressed and the boy was referred to our department for complex evaluation and assessment. At this time, we observed a notable psychomotor retardation, significant central hypotonia, limited spontaneous movement, poor eye contact, no reaction on noise, significant failure to thrive (with only 90 g gain in 2 weeks). Arthrogenic contractures limiting the range of joins mobility were observed mainly in knee joints, ankle joints and elbow joints. His dysmorphic features included dolichocephaly, bossing forehead, dysmorphic low set ears (Figure 1A), enlarged fontanelle, wider philtrum, broad nasal root, prominent nares, hypertelorism, retrognathia (Figure 1B), inverted nipples (Figure 1C), abnormal fat distribution over his thighs, buttocks and suprapubic regions (Figure 1D,E), pilonidal sinus and hammertoes (Figure 1F).

Laboratory analyses at that time revealed an altered biochemical profile with findings of hypoproteinemia, hypoalbuminemia (23.1 g/l), severe combined coagulopathy with coagulation and anti-coagulation pathways alterations (aPTT 1.86R, INR 1.3, antithrombin III 20%, Factor IX 31%, fibrinogen 1.72 g/l, severe deficiency of Factor XI 5%, D-Dimer: 1.37 mg/l, Factor VII 66% and protein C bellow 5%). Other laboratory findings included mild hepatopathy with elevated levels of AST (140.96 IU/l), ALT (77.11 IU/l) and ALP (611.45 IU/l), mild elevation of lactate dehydrogenase, creatine kinase, and TSH. Infectious disease screening was negative.

Abdominal ultrasonography showed hyperechogenicity of kidneys, mild renal pelvis dilatation, ascites and small pleural effusion. There were no laboratory signs of nephropathy. The patient underwent cardiac examination. Cardiac anatomy and function were normal; however, a chronic pericardial effusion was detected by echocardiography. Initially mild to moderate pericardial effusion slowly progressed despite the administration of the diuretic (furosemide at 1mg/kg/day). The patient showed also intermittent eyelid edema and swelling of the arms and legs. Repeated infusions of albumin and plasma substitutes always led to the improvement of clinical symptoms including partial regression of pericardial effusion and peripheral edema.

Clinical symptoms and laboratory findings led to the strong suspicion of PMM2-CDG. Screening for CDG performed by the use of isoelectric focusing of serum transferrin showed pronounced increases in disialotransferrin and asialotransferin with a corresponding reduction in tetrasialotransferrin. This pattern supports the diagnosis of PMM2-CDG.

**FIGURE 2:**
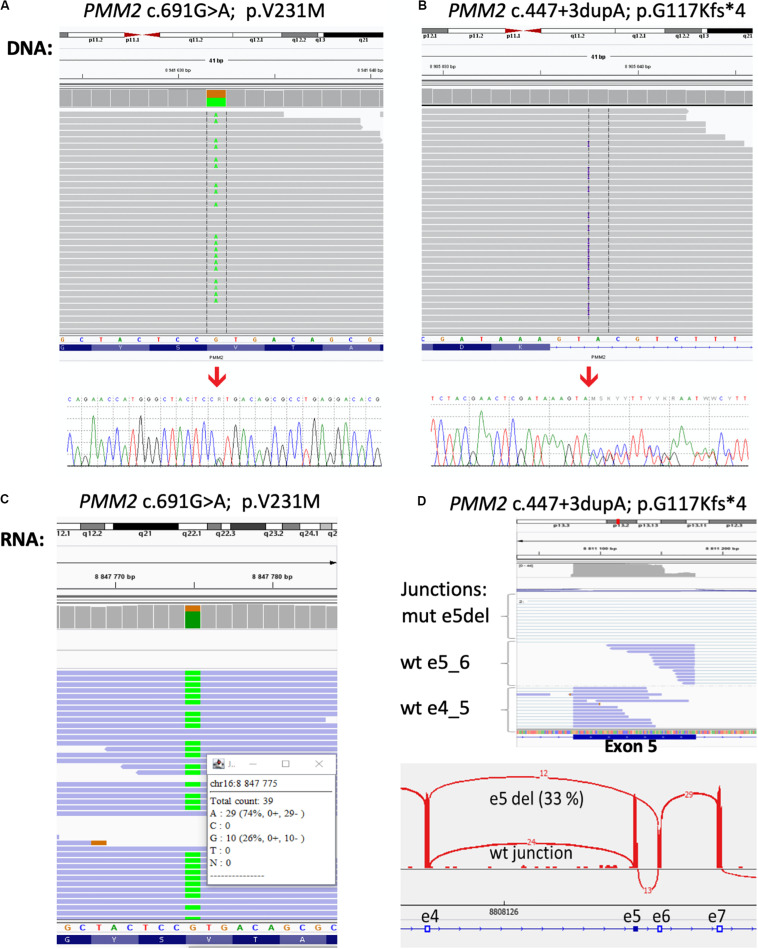
Results of the DNA and RNA sequencing. Molecular analysis presenting variant c.691G > A/p.Val231Met **(A)** and variant c.447 + 3dupA in the PMM2 gene **(B)** in the patient’s DNA from exome sequencing in Integrative Genomics Viewer (IGV) and Sanger sequencing analysis (bottom part). At RNA level, over 70% of mRNA was produced from c.691A > G allele **(C)** and the proportion of exon five skipping was over 30% of all transcripts **(D)**.

To further confirm the genetic cause of the disease exome sequencing was performed. Library for whole-exome capture and sequencing was prepared using TruSeq Exome Kit. Prepared library was loaded onto NextSeq 500/550 Mid Output Kitv2.5 (150 cycles) and sequenced on the NextSeq 500 instrument (all Illumina, CA, United States). Sequencing coverage for exomes was > 20 × at > 90% of captured regions. The variants were filtered to include those with low frequency and a predicted effect on the protein. The frequency-filter removed variants with prevalence > 1% in GnomAD or 1000 Genomes databases. The predicted effect filter excluded synonymous and non-coding variants unless they were located within 20 bp from the end of an exon. Variants annotated in ClinVar as pathogenic/likely pathogenic were retained regardless of their frequency/function. The variants found in the proband’s sample that were considered to be significant to CDG were c.691G > A/p.Val231Met in the *PMM2* gene known to be disease-causing (Matthijs et al., 1997;) and classified as class 5-pathogenic accordingly to ACMG/AMG system (Richards et al., 2015) and novel variant c.447 + 3dupA in the PMM2 gene (NM_000303.2, intron 5). We did not detect any other class 5 or 4 variants in the genes for which incidental findings are reported based on the ACMG guidelines. Confirming the results of the exome sequencing, the Sanger sequencing was done in the proband and his parents. As it was expected, results confirmed both variants in the proband and showed that c.691G > A/p.Val231Met variant was inherited from father (Figure 2A) and c.447 + 3dupA variant from mother (Figure 2B), presenting the typical autosomal recessive mode of inheritance.

The c.447 + 3dupA variant causes single-nucleotide duplication of the third intronic nucleotide after exon 5. Three different bioinformatic tools (MaxEntScan, NNSPLICE, and SpliceSiteFinder) integrated in the Alamut Visual 2.11 software predict a loss of donor splice site (Interactive Biosoftware, France) with confidence 75%. To confirm the impact of this variant to the mRNA splicing process, we have performed RNA sequencing. Total RNA from peripheral leukocytes was used to prepare the sequencing library with NEBNext Ultra II Directional Library Prep Kit (New England Biolabs, MA, United States). The library was loaded onto NextSeq 500/550 High Output Kit (75 cycles) and sequenced on the NextSeq 500 instrument (both – Illumina, CA, United States). The results of RNA sequencing were visualized by the IGV software^1^ using Sashimi plots for individual exon–exon splicing events present in the transcript. We have confirmed that c. 447 + 3dupA variant causes a loss of donor splice site, leads to exon five skipping (r.348_447del100), and causes premature termination of translation and protein truncation (p.G117Kfs^∗^4; Figure 2C,D), and therefore, it can be classified as a class 4—likely pathogenic.

The diagnosis of PMM2-CDG was confirmed by clinical, biochemical, and genetic findings.

**FIGURE 3:**
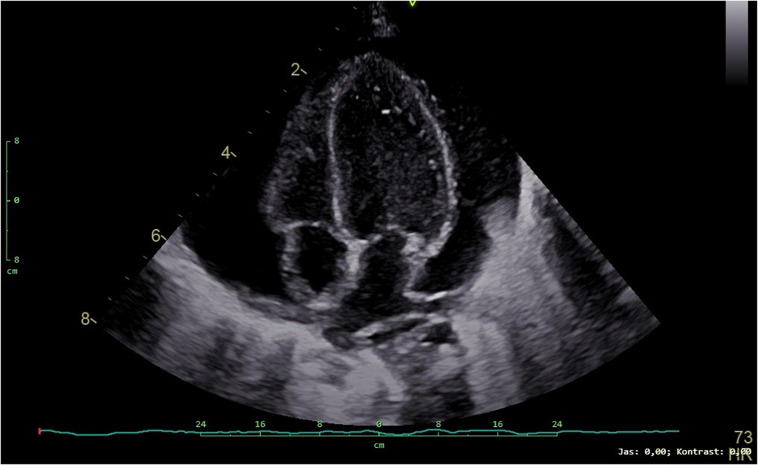
Transthoracic echocardiogram (4-chamber view) showing a large pericardial effusion in our patient.

At the age of 3 months, the patient was admitted to the intensive care unit for low food intake and clinical deterioration. He presented significant failure to thrive with weight being 3060 g (below 0.1 percentile, SD −5.15), length of 52 cm (below 0.1 percentile, SD −5.31), and fronto-occipital circumference 38.5 cm (below 0.1 percentile, SD −3.93). The child showed tachypnea and dyspnea becoming worse with feeding. Pericardial effusion further progressed (Figure 3) and finally required pericardiocentesis. Pericardial fluid was considered to be a transudate and serosanguineous. The patient was clinically deteriorating, respiratory and circulatory failure progressed. Despite all efforts, the patient died due to obstructive cardiogenic shock at the age of 3 months.

## Discussion

High clinical heterogeneity of various CDG types, and even within one particular CDG type, is considered to be the main reason behind underdiagnosis or late diagnosis of this disease (Giurgea et al., 2005). Alterations in glycosylation patterns are usually determined by isoelectric focusing of serum transferrin and apolipoprotein C III. The analytical performance of the isoelectric focusing of serum transferrin, which is a biomarker of N-glycosylation, is not satisfactory with only 60% of CDG cases being accurately found to be positive. Further, this test cannot reliably identify the subtype of CDG, and not all types can be detected by this approach (Altassan et al., 2019). A similar test to help diagnose O-linked disorders and combined N- and O-glycosylation CDGs is performed on apolipoprotein-CIII which has a single O-linked glycan which only captures abnormalities of core-1mucin type O-glycosylation. The diagnosis of PMM2-CDG can then be confirmed by measurement of PMM2 activity in either peripheral blood leukocytes or cultured skin fibroblasts (Francisco et al., 2019). The majority of PMM2-CDG patients have residual PMM2 activity below 10%, whereas in their asymptomatic parents the enzyme activity is about 50% (Francisco et al., 2019).

The variants found in the patient DNA by exome sequencing and confirmed by Sanger sequencing were a common variant c.691G > A/p.Val231Met (Altassan et al., 2019) and a novel variant c.447 + 3dupA in the *PMM2* gene. The novel mutation, inherited from mother, causes single-nucleotide duplication of the third intronic nucleotide after exon 5 and is predicted to result in loss of donor splice site indicating a novel splicing variant of the *PMM2* gene. Although three predictive algorithms predict the role of this novel variant in splicing, it has to be further experimentally confirmed to have predicted impact on the *PMM2* transcript splicing.

The variant p.Val231Met, despite being the second most frequent mutation in the *PMM2* gene with a prevalence of 10%, has only been reported so far as a compound heterozygote (Altassan et al., 2019) as in our case, where this allele was inherited from father. V231 is in the interior of the core domain, and a mutation in this residue is detrimental to its native protein structure (Silvaggi et al., 2006; Citro et al., 2018). The folding and stability defect of the Val231Met allele contributes to its reported reduced *in vitro* enzymatic activity of 38.5% (Kjaergaard et al., 1999).

Most of the PMM2-CDG patients are born with hypotonia, craniofacial dysmorphisms, and strabismus. Common are inverted nipples and a characteristic distribution of the fat pads especially in the gluteal and suprapubic regions and thighs. Clinical spectrum of PMM2-CDG further includes psychomotor retardation and mild to severe intellectual disability. PMM2-CDG patients typically show different degrees of cerebellar atrophy on MRI, mostly vermian atrophy (Serrano et al., 2015). In our patient, MRI was not feasible due to clinical deterioration. Cerebellar atrophy was not confirmed post-mortem, because parents declined an autopsy. In the small percentage of the patients, there is a prenatal generalized edema and abnormal accumulation of fluid in two or more fetal compartments, hydrops fetalis, or accumulation of fluid in one specific compartment as in our patient with prenatal pericardial effusion.

Up to 15% of non-immune hydrops fetalis cases may be due to inborn errors of metabolism, and a large proportion of cases linked to metabolic disorders remains undiagnosed. The pathophysiology of fetal hydrops, edema, and pericardial effusions in inherited metabolic diseases is not fully understood. The fluid accumulation is due to an unbalanced interstitial fluid production and lymphatic return. The etiology of these symptoms in CDG appears to be multifactorial, frequently occurring due to a decreased plasma albumin concentration secondary to enteral and renal protein loss and a decreased synthetic function of the liver (Truin et al., 2008). There are additional factors leading to fluid leakage to pericardial and peritoneal spaces. Focal mixed inflammatory changes with mesothelial proliferation and a damaged pericardial protein barrier have been suggested in PMM2-CDG patients previously (Kristiansson et al., 1998). Truin et al. (2008) described three patients with severe *PMM2* mutations, who developed life-threatening accumulation of pericardial and abdominal fluids. In two cases this severe extravascular fluid accumulation progressed to decompensation and death.

Abnormal glycosylation of cell surface proteins in PMM2-CDG patients may result in disequilibrium of normal fluid balance and protein transport through the pericardial and peritoneal membranes and may cause life-threatening complications (Isikay et al., 2014).

## Conclusion

In this case report, the patient’s non-specific but uncommon findings of pericardial effusion, inverted nipples, fat pads, and combined coagulopathy pointed us toward an early clinical suspicion of a PMM2-CDG disease. Consequent targeted biochemical analysis and genetic findings led to the confirmation of the PMM2-CDG. We identified one common pathogenic variant and one novel variant predicted to result in loss of donor splice site, leading to exon five skipping and causing premature termination of translation to the protein. There is no curative therapy for the PMM2-CDG at the moment, but other supportive care options were available to be offered. The definite diagnosis of PMM2-CDG can also assist in the process of genetic counseling, family planning, and preimplantation genetic diagnosis.

## Data Availability Statement

The raw data supporting the conclusions of this article will be made available by the authors, without undue reservation.

## Ethics Statement

The study was reviewed and approved by the Ethics Committee of University Hospital Brno. Written informed consent to participate in this study was provided by the participants’ legal guardian. Written informed consent was obtained from the minor(s)’ legal guardian/next of kin for the publication of any potentially identifiable images or data included in this article.

## Author Contributions

KS, OS, and DP designed the study and drafted the manuscript. HN, PV, PK, and OS performed the genetic analysis and evaluation of variants. KS, JT, DP, TH, PS, HJ, and PJ conducted the clinical evaluations. HH and TH performed the biochemical analysis. All authors approved the final manuscript.

## Conflict of Interest

The authors declare that the research was conducted in the absence of any commercial or financial relationships that could be construed as a potential conflict of interest.

## Footnotes

1 http://software.broadinstitute.org/software/igv/
